# Colours of the mind: A qualitative study exploring the relationship between neurodivergence and creativity

**DOI:** 10.1371/journal.pone.0359079

**Published:** 2026-10-28

**Authors:** Ben Erin, Sebastian C. K. Shaw, Lisa Quadt, Jessica A. Eccles

**Affiliations:** 1 Department of Clinical Neuroscience, Brighton and Sussex Medical School, Brighton, East Sussex, United Kingdom; 2 Department of Medical Education, Brighton and Sussex Medical School, Brighton, East Sussex, United Kingdom; 3 Sussex Partnership NHS Foundation Trust, Worthing, East Sussex, United Kingdom; University of North Carolina at Chapel Hill, UNITED STATES OF AMERICA

## Abstract

The neurodiversity movement advocates for a societal framework that recognises neurodivergence as a difference, rather than a disorder. Despite the advocacy efforts, neurodivergent people still face issues with their physical and mental health at higher rates than the general population. Some scholars argue that these disproportionate rates originate from stress resulting from discrimination and societal stigma faced by neurodivergent people. This study aims to challenge these societal views through exploring one of the reported strengths of being neurodivergent: creativity. This study aims to explore *why* neurodivergent populations may be creative. To research this complex relationship between being neurodivergence and creativity a qualitative study was conducted. Ten neurodivergent adults who had studied or were currently studying a creative degree were interviewed on Zoom, exploring their beliefs about this relationship. An inductive thematic analysis was then carried out. The analysis gave rise to three themes: *(1) The Construct of Creativity, (2) Resilience, & (3) Empowerment*. These themes provided hypotheses for understanding the connection between neurodivergence and creativity. The results provided two hypotheses. The ‘innate trait hypothesis’ posits that traits thought to be intrinsic to neurodivergence may be beneficial in fostering creativity. The innate traits that arose were *divergent thinking*, *monotropism* and *communication differences.* The ‘social isolation hypothesis’ posits that the discrimination that neurodivergent people face leads to profound isolation and in some cases mental health challenges. Neurodivergent people may then use creativity as a method for regulating their emotions, providing solace from distress. Moreover, the isolation they experience provides an opportunity for introspection, which fuels creativity. Our results highlight that some neurodivergent traits, when placed within a creative context, can become strengths. This highlights the possibility that the pathologisation of neurodivergent traits is not just damaging but also invalid, with the reality being much more nuanced.

## Introduction

‘Neurodiversity’, a term developed collectively among autistic activist communities, refers to the natural diversity of neurological traits in the entire human population, emphasising that the everyone is different with unique abilities and needs [1,2]. This concept contributed to the birth of the neurodiversity movement, advocating for a society that recognises and respects the diverse neurological makeup of all individuals [3]. This movement frames neurodivergence as a difference – the strengths and challenges of neurodivergent people because of this difference will be down to external rather than internal factors [3,4]. Neurodiversity is often a term misused as a synonym for neurodivergence. Neurodivergence refers to a mind that functions in a way which diverges significantly from the societal conceptions of ‘normal’; someone who thinks differently to the majority [5]. Neurodivergence encompasses neurominority populations such as autistic, ADHD, dyspraxic, dyslexic, dyscalculic, and Tourette’s [5].

Neurodivergent people face mental health issues at a higher rate to the general population [6,7]. Studies have found that both autistic and ADHD adults experience higher levels of suicidal ideation and behaviour than the general population [8,9]. These health inequities also extend into the physical health domain, with studies finding a whole host of medical conditions including diabetes, hypertension and obesity being more common in autistic and ADHD people in comparison to the general population [10–12]. Research into understanding the contextual factors that contribute to these poorer outcomes is still emerging, however using the ‘social model approach’, it can be argued that these health disparities are not due to characteristics innate to neurodivergence, but instead arise from barriers imposed by societal norms [13]. These barriers extend to healthcare, influencing both access to appropriate services and interactions with healthcare professionals, which in turn can limit access to timely, evidence-based, affirming, and neurodivergence-sensitive care. Multiple studies have described barriers to accessing healthcare imposed on autistic people [14,15]. Autistic people commonly cite challenges in communicating with healthcare staff as one of the most significant barriers they face [16]. Furthermore, minority stress theory, originally developed to explain health disparities among sexual and gender minority populations, proposes that chronic exposure to stigma, discrimination, and prejudice contributes to poorer health outcomes [17]. More recently, this framework has been applied to neurodivergence, suggesting that many of the health inequalities experienced by neurodivergent people arise not solely from neurodivergence itself, but from the cumulative effects of social stigma, discrimination, and exclusion [17]. This is illustrated by the fact that social isolation is more common in both ADHD and autistic people than the general population, which is thought to contribute to the disproportionate rates of mental health issues [18,19]. Furthermore, one study found that children with neurodivergent traits at the age of 7 and 9 were twice as likely to experience chronic disabling fatigue by the age of 18 [20]. These health disparities highlight the need for societal attitudes surrounding neurodivergent people to be challenged.

When examining the discriminatory societal attitudes facing neurodivergent people, it is essential to consider the role that medical institutions may play in this. Historically, the inclusion of homosexuality in the Diagnostic and Statistical Manual (DSM) between 1952 and 1973 led to medical professionals pathologising and classifying homosexuality as a mental disorder, which contributed to the stigmatisation of the LGBTQ+ community [21]. Similarly, discussions of neurodivergence have increasingly considered how medical models, when focused predominantly on deficits, may inadvertently reinforce stigma by framing neurodivergent traits primarily as traits in need of correction rather than as forms of human diversity [22,23]. Furthermore, epidemiological studies have concluded that some forms of neurodivergence, such as ADHD and autism are currently underdiagnosed [24,25]. Through adopting a more balanced conceptualisation of neurodivergence, this may help reduce self-stigma and encourage greater engagement with diagnostic and support services. As such, it can be argued that it is essential to move away from the current deficits-based approaches to screening and diagnosis of neurodivergence and begin to move towards a more balanced approach to diagnostic criteria, recognising both the contextual strengths and challenges of being neurodivergent.

Creativity has been identified in the literature as a characteristic associated with some forms of neurodivergence. Anecdotally, some neurodivergent individuals also describe creativity as an important aspect of their lived experience. Creativity is a subjective concept, which is challenging to define due to its multidimensional nature. It is for this reason that there are often struggles in measuring this construct [26]. Despite this, quantitative attempts to measure the relationship between neurodivergence and creativity have been attempted, which have yielded mixed results. In the case of autistic people, one systematic review concluded that there are fewer creative autistic people compared to the general population, but these people did seem to reach higher levels of originality than the comparison groups [27]. This raises the implications of the subjective nature of creativity since some may argue that originality is intertwined with creativity as a concept. Similarly with ADHD people, one review shows a link between ADHD and creativity, but this was more specific for adults and divergent thinking [28]. In the case of dyslexic people studies find inconsistent and unclear results [29,30]. Finally, in the case of Tourette’s syndrome, the relationship is again unclear. Some studies do find suggestions of a link, but often the results are not statistically significant [31]. These inconsistent findings underscore the complexity of the relationship between neurodivergence and creativity. However, the research methodologies employed in some studies may contribute to these mixed results. One group of researchers has pointed out that the creativity measures commonly used may not be inclusive or appropriate for neurodivergent individuals, particularly autistic people and those with ADHD. [32]. Their creativity is not being allowed to unfold on their own terms – neurodivergent creativity does not conform to standard expectations, making it challenging to assess [32]. These researchers have advocated for a more inclusive creativity measure which can capture this proposed relationship [32]. Building on this work, the present study adopts a qualitative approach to explore how these people understand and describe their own creativity in relation to their neurodivergent experience.

### Aims

The aims of this project are not to formulate a generalisable model explaining why neurodivergent people may be more creative nor is it to compare their creativity to neurotypical people. Instead, it aims to centralise the experiences of neurodivergent creative people, to propose various hypotheses behind their creativity. Moreover, it aims to explore the individual experiences of neurodivergent adults in relation to their personal relationships with creativity, while allowing for their voices and expertise to be heard in research. No prior studies have taken such an approach to this relationship.

## Materials and methods

### Paradigmatic and methodological considerations

This is a qualitative study, drawing on critical realist ontological and epistemological underpinnings. Put simply, critical realism posits that reality is objective and independent of human perception – a realist ontological stance [31]. However, unlike the more traditional positivism, critical realism also acknowledges the influence of human perception on the creation of knowledge – a subjective epistemological stance [33]. In practice, critical realism therefore embraces a range of potential research approaches, including qualitative. An important consideration, however, differentiating a critical realist approach from more interpretivist qualitative research is the belief that there is a right answer out there to be found (realism) [33]. This results in methods with a slightly more objective style than might be seen within interpretivism or social constructionism, for example. In practice, this can include concepts like consideration of bias or researcher influence on the study.

A qualitative approach is well suited to our research aims for various reasons. Both neurodivergence and creativity are subjective constructs. Neurodivergence has a fluctuating definition, which relies on societal conceptions of the ‘normal’ brain. What it means to be neurodivergent will differ between individuals and change over time. It is a similar case with creativity, which is another concept that is challenging to define. Through using qualitative methods, we will attempt to embrace this subjectivity, without imposing any definitions on any participants.

### Positionality and justice

Within qualitative research it is important to consider one’s own positionality in relation to the study [34]. When researching marginalised groups like neurodivergent people it is all the more important to consider such things, as a matter of justice. There is a contested history of conducting research ‘on’ neurodivergent people and not with them [35,36]. This has fostered an epistemic injustice, whereby the credible status of neurodivergent people as creators of knowledge about themselves has been diminished [15]. It is therefore vital that neurodivergent voices are central to the creation of new knowledge in this area, to help overcome decades of these previously entrenched practices. This not only helps neurodivergent voices to be heard in places of influence, but also facilitates research aligned with neurodivergent priorities [37]. In the case of this study, we also argue the only current experts in this field of neurodivergence and creativity will be neurodivergent people themselves. To that end, our authorship team includes neurodivergent representation, providing an insider perspective. Furthermore, our choice of methodology allows us to centre neurodivergent voices and expertise to formulate our subsequent hypotheses for understanding the relationship between neurodivergence and creativity.

### Participants & recruitment

This study aimed to recruit 8–12 neurodivergent people who were studying, or historically had studied, a creative degree programme. Each participant needed to be over 18 years of age and formally diagnosed as neurodivergent. For the purpose of this study, we considered this to include: autism, ADHD, dyslexia, dyspraxia, dyscalculia, dysgraphia, or Tourette’s.

Recruitment was carried out via the distribution of a poster (appendix 1) advertising the study via the social media platform ‘X’ (previously known as ‘Twitter’). The poster advertised the inclusion criteria and stated the nature of the study would be an interview that roughly lasts 30 minutes to 1 hour with the lead researcher (BE). The poster advised potential participants to email BE if they were interested in taking part. A financial incentive was also offered through a £16 gift pay voucher to aid recruitment.

### Procedure

Once a potential participant emailed BE, they were emailed a Participant Information Sheet, which detailed more information about the study. After reading this and having the opportunity to ask any questions, if a potential participant was still interested, they would be emailed a consent form. Signed informed consent was received from each participant prior to interviews. The recruitment period spanned from November to December 2023, with all interviews taking place in December 2023. Informed consent was checked again verbally prior to the start of the interview and participants were once more offered the opportunity to ask any questions.

An interview topic guide (appendix 2) was produced by BE. Participants were interviewed in a semi-structured manner over Zoom. In planning these interviews, it was agreed that should participants express a difficulty understanding any questions, BE would offer to type these into the chat function on Zoom. He also ensured that participants were aware there was no time pressure to answer any questions and that they were welcome to answer questions using the chat function if they preferred. Broad topics which were explored in the interview are described below.

1. What is means to be neurodivergent.
2. What it means to be creative.
3. Why there might be a relationship between being neurodivergent and creativity.

The interviews were audio-recorded and transcribed via Zoom’s internal software. The transcriptions were carefully reviewed by BE to ensure they were accurate and were then stored in a password protected folder on the University OneDrive. The audio recordings were permanently deleted after this transcription was complete.

### Data analysis

The data were analysed utilising the inductive thematic analysis approach of Braun and Clarke [38,39]. BE initially familiarised himself with the data by thoroughly reading and re-reading the interview transcripts. He then organised the data in a systematic manner, generating initial codes guided by the research question to make the data more manageable. This was achieved using the highlighting and commenting features of Microsoft Word. A second researcher (LQ) also independently coded the transcripts and both researchers (BE and LQ) agreed on the final codes that emerged from the analysis. Next, these initial codes were analysed and organised these into themes that captured something significant in terms of the research question. Quotations which were representable of these initial themes were then selected. Themes were continuously reviewed and refined to ensure they accurately reflected and were supported by the data. When overlapping themes were identified, they were consolidated into a single overarching theme with multiple sub-themes. The analysis was conducted without any preconceived notions regarding the relationship between neurodivergence and creativity, allowing the data itself to guide the findings- characteristic of an inductive thematic analysis [37]. To ensure rigor in the analysis, drawing on the study’s critical realist assertions, the researchers engaged in self-reflection to minimise observer bias and keep the participants’ voices central. With this in mind, after an initial analysis, BE then engaged in iterative discussions with SS about the evolving analysis, which helped to further refine it over time.

Following the thematic analysis, BE aimed to integrate the findings with existing literature to develop comprehensive hypotheses which can help generate understanding for the relationship between neurodivergence and creativity. To achieve this, BE conducted an extensive literature search to align the thematic analysis results with, where possible, current knowledge in the relevant fields. Through utilising these methods this study takes a critical realist approach, as aforementioned, arguing for the role that individual subjective interpretation takes in objective reality [31]. It views the relationship between neurodivergence and creativity as an objective phenomenon, arguing it is possible to identify the mechanisms that lie beneath its existence. However, it also acknowledges that individual subjective interpretation plays a role in defining this reality arguing that separation of the changing nature of social reality from this relationship is not possible. This study employs a flexible approach to methodology. This study utilises qualitative methods, which allow for the subjective reality of creativity to be acknowledged and for the exploration of individual experiences and perceptions. Then through building on this analysis through incorporating any relevant literature on the subject it aims to recognise the objective reality behind this relationship. This led to the formation of two hypotheses which are described in detail in the ‘Discussion’ section of this paper.

## Results

Ten people were recruited to participate in this study, with over 50 expressing interest in taking part. The first participants who emailed were offered a place in the study. If they chose not to participate, the next person who emailed would be offered the opportunity instead. The 10 participants were each given a pseudonym as shown in Table 1.

**Table 1:** Demographic information of our participants.

Pseudonym	Age range	Creative course	Neurodivergent identity
Amanda	20-25	Fine art	Autistic
Brian	*	Fashion design	Autistic
Caleb	*	Creative arts	ADHD
Delilah	20-25	Fine art	Autistic
Eddie	20-25	Music	Autistic
Fabian	20-25	Fine art	Autistic
Grace	20-25	Animation	AuDHD*
Hana	26-30	Creative writing	Autistic
India	26-30	Fine art	Autistic
Jasmine	20-25	Music	Autistic

*Participants chose not to disclose their age for the study

**AuDHD represents the coexistence of autism and ADHD

The thematic analysis revealed three themes: (*1) The Construct of Creativity, (2) Resilience and (3) Empowerment*. *The Construct of Creativity* outlines what creativity means to neurodivergent people *(1.1)* before illustrating how neurodivergent creativity is considered different to neurotypical creativity by some of our participants *(1.2).* Subsequent themes build on this to explore how elements of a neurodivergent identity foster creativity. *Resilience* illustrates how creativity can provide an opportunity for neurodivergent people to overcome their experiences with isolation (*2.1*), challenges with regulating their emotions (*2.2*) and communication differences (*2.3*). *Empowerment* investigates an alternative angle, recognising how creativity may empower neurodivergent people fostering a sense of agency and self-worth. Creativity allowed our participants to celebrate their differences in thinking *(3.1)* and find value in their neurodivergent identities *(3.2).* The results of this thematic analysis are represented graphically in Fig 1.

**Fig 1:**
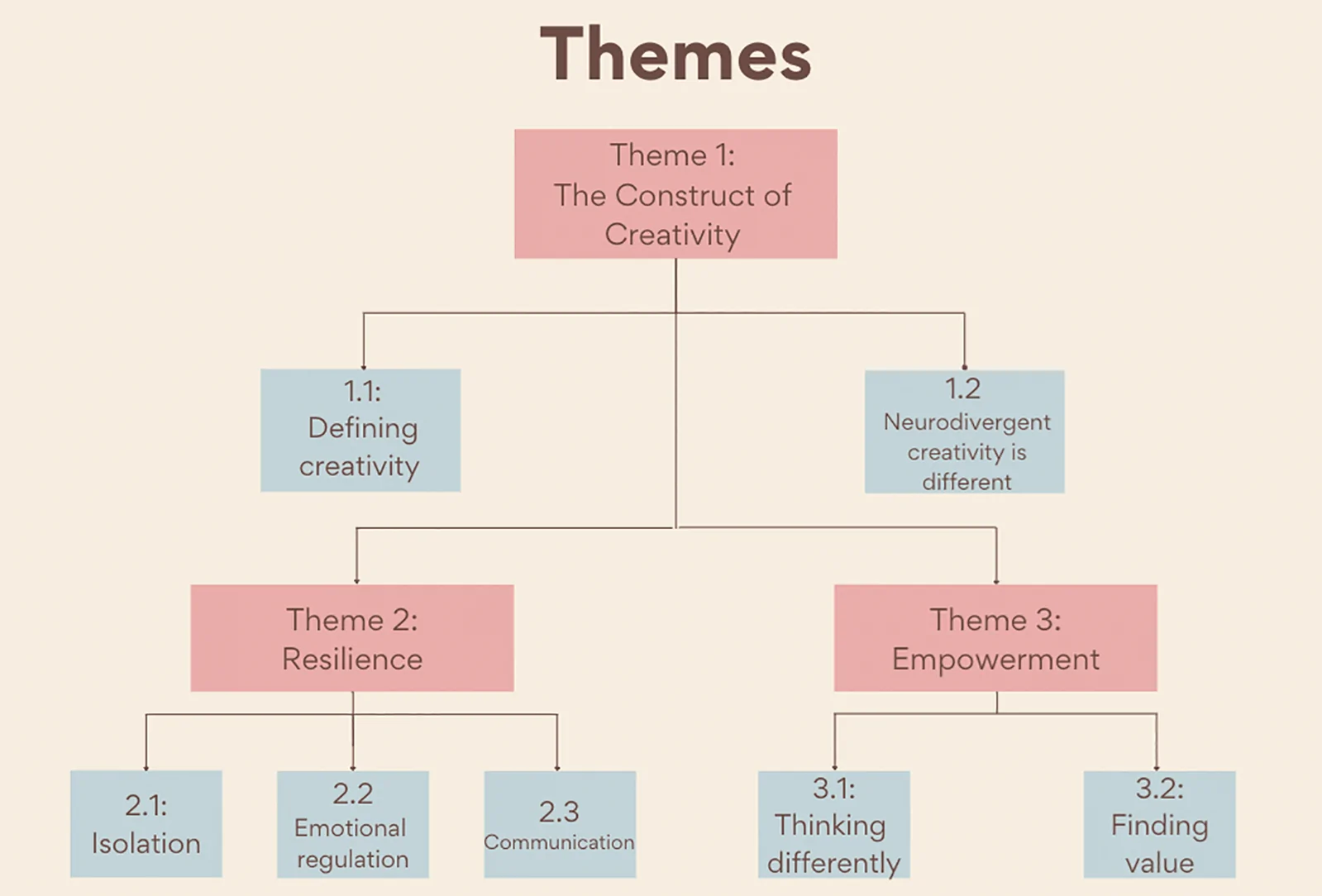
Overview of themes and sub-themes from the analysis.

### Theme 1: The construct of creativity

Creativity is a multifaceted construct that can be defined and interpreted in numerous ways. This theme explores the differing meanings and expressions that creativity holds for the neurodivergent participants of our study. Sub-theme *defining creativity (1.1)* explores how creativity is perceived by our participants while sub-theme *neurodivergent creativity is different (1.2)* explores how neurodivergent creativity is perceived as distinct from that of neurotypical individuals.

#### 1.1 Defining creativity.

Within our participants the meaning of creativity manifested in diverse ways. Some felt the essence of creativity lies in the ability to innovate and produce something new or unique.

*“I feel like when people think of creative subjects, they only think of just like art and music. But I think it's anything that, you know, requires, you know, invention or creation of something new.” – Grace*

*“Being creative to me definitely means being different, adding more beauty or fashion to whatever I do to make the outcome different from the norm.”- Caleb*

Other participants considered creativity to relate more closely to diversity in thinking and the ability to approach situations from multiple angles.

*“Creativity to me is being able to, being able to reflect around that thing from diverse ways, not just one perspective”-Hana*

*“I actually think you are being creative means… actually seeing things from other people's own perspectives.”- Delilah*

Passion also emerged as a significant component of creativity for some participants, underscoring the idea that genuine creativity is fuelled by intense enthusiasm for one’s work.

*“All I'm saying is that creativity can only come from where there is passion. Where there is love for creativity.”- India*

#### 1.2 Neurodivergent creativity is different.

One significant observation from our participants was the belief that neurodivergent creativity is distinct from that of neurotypical individuals.

*“Autistic people are genuinely creative on their own, in their own way. We're not creative based on how people want us to be… We are creative based on our experiences, based on how we understand our autistic experience and language.”-Hana*

They considered this to be the case through both their own experiences and their observations of neurotypical others.

*“There are things that I see in depth, that other [neurotypical] people I work with don't see when it comes to creative mindsets.”- Jasmine*

In the case of our participants, their own specific creative interests were diverse. Examples included drawing, painting and video creation.

*“I had passion for understanding music and being able to portray it in crafts, visual arts, video creation, and paintings.” – Caleb*

*“I've always loved to draw an image of myself then to see a better version of myself and also see a better version of people”- India*

Our participants recurrently reported the intense passion they felt for their creative pursuits. They felt that their neurodivergent attributes, such as hyperfocus, enhanced their creativity.

*“I have this intense passion for music that helped me immerse myself and helped in heightening my creative potential.”- Jasmine*

This section has explored creativity as a complex and deeply personal construct, highlighting the unique ways neurodivergent individuals perceive and express their creativity. The subsequent sections will move to explore some of the unique aspects of neurodivergence that may lead to developing creative traits.

### Theme 2: Resilience

The theme of resilience emphasises how creativity can provide neurodivergent people with a way to navigate and overcome the various challenges associated with their life experiences*.* This theme illuminates our participants’ capacity to navigate and adapt to the challenges stemming from societal attitudes towards their neurodivergent identities. Within this theme, there are sub-themes of *isolation (2.1)*, *emotional regulation (2.2)* and *expression (2.3)*.

#### 2.1 Isolation.

One of the reasons participants had to build resilience was due to their experiences of isolation – a sentiment that resonated with all 10 participants. These participants discussed the profound negative impact of social isolation.

*“So I really wish people would understand that autism is not something you isolate people about. You make them feel less of themselves. Any autistic person is the same as you, who is a non-autistic person. All we need is just a double care, double love, double attention.” -Hana*

Many participants, however, expressed that this isolation afforded them the freedom and privacy needed to deeply reflect on various aspects of life, enabling them to explore and articulate their thoughts through creative pursuits.

*“But I feel like there is this, there is this bond between creativity and autism because this is you being soaked in your own world, being kept in your own world to reflect, to think.”- Hana*

Some participants found that their creativity flourished amidst isolation, allowing them to view this not as a barrier, but instead as a source of strength.

*“Yes, the strength of my art is the ability to isolate. I mean the isolation part has made me be able to stay on my own giving me the freedom and privacy to reflect on several things in life and then after reflection I go about exploring and then writing down”- India*

#### 2.2 Emotional regulation.

Furthermore, in addition to isolation, many participants in this study appeared to build resilience through their experiences with regulating their emotions. They recognised that their mental health challenges were often exacerbated by societal attitudes towards neurodivergent people. Participants frequently discussed their mental health struggles and considered creative expression to be a sanctuary for managing and transcending overwhelming emotions.

*“The passion [for music] has come from the fact that whenever I was troubled and I listened to music, it takes me to a far place… where I forget everything that got me worried. It gives me this peace; it gives me this calmness and it's more conducive for my mental health.”- Jasmine*

*“[creativity] is a distraction to the fact that I am autistic and has made my mental health sound and stable because if you don't have anything that you're passionate about then you wouldn't have anywhere or anything to channel all your energy and attention to and you would still remain in a space where you feel isolated and you feel down.”- India*

#### 2.3 Communication.

Finally, the sub-theme of communication emerged prominently within the participants’ narratives. All 10 participants referred to their struggles with social communication with others, many of these indicating that this was a distressing and challenging experience.

*“I panic… I can't keep association, I can't keep conversations, I can't maintain eye contact and all of that. So, it's sometimes difficult. Because a lot of people might think that you're not bold enough or you're less than them.”- Caleb*

One significant resonance among certain participants was the realisation that, despite grappling with communication challenges, their interest in socialising remained present.

*“[Struggling with communication] does not mean that we are not interested in socialising. It simply means that I maybe prefer another form of communication, or that I require extra support to engage socially.”- Jasmine*

Many of the participants expressed a sense of comfort and agency when expressing themselves creatively as an alternative to ‘traditional’ forms of interaction. These alternatives forms of creative communication included expression through music, painting or creatively writing.

*“Because I don't want to interact with people because I have some, you know, when I want to talk, I just paint it”- Amanda*

The communication differences experienced by some participants provided them an opportunity to express themselves creatively. This creativity offered a meaningful avenue for self-expression, highlighting the importance of recognising and supporting diverse communication preferences in neurodivergent people.

### Theme 3: Empowerment

Where *theme 2* provided an insight into how creativity helped to manage some challenges faced by our participants, this theme now examines how creativity empowered them to view their neurodivergent identities positively. The narratives of participants in this analysis revealed empowerment as a central theme, encompassing the subthemes of *Thinking Differently (2.1)* and *Finding Value (2.2).*

#### 3.1 Thinking differently.

One way in which the participants expressed that their creativity empowered them was through their ability to think differently. Several participants articulated that their thought processes diverged from those of people who are not neurodivergent, noting the advantages this brings to their creative endeavours. Creativity seemed to empower our participants to perceive their unique way of thinking positively.

*“Because our brains are inherently like wired differently that we end up just thinking of things that other people wouldn't “- Grace*

*“This attention to this detail can be advantageous… as it allows us people with autism to observe unique aspects of our surroundings and make unique connections.”- Jasmine*

As outlined in *theme 1*, intense passion was something that many participants instigated was advantageous for creative process. Some participants argued that this intense passion was innate to autism.

*“I feel like passion is like something that is very innate in autistic people. And even if it's not like, artwork, they're creative in other ways”- Grace*

Building on this, some participants mentioned that this intense passion was related to the neurodivergent trait of hyperfocus. This was viewed as a strength in creative processes, allowing them to express their intense passions and interests through creativity. For some, creativity provided a conducive outlet to fully immerse themselves in their interests and build connections through this.

*“We don't want to just like casually like watch and enjoy, but like we want to take part in it. Autistic people… can be so hyper focused on their passions that they will throw themselves into it completely and find creative careers that way.”- Grace*

*“Well autism had made me direct and channel all my attention to drawing”-India*

#### 3.2 Finding value.

Our participants did not solely find empowerment through recognising their different types of thinking. Another sub-theme focused on neurodivergent people utilising creativity to discover their intrinsic worth in a society that often marginalised or stigmatised them. Our participants’ responses frequently illustrated otherwise harrowing effects of societal stigma.

*“I've always seen myself as someone who might be neglected in the society because of the way I feel at times, because of the way I reason at times, because of my lack of understanding people, my lack of communication with people, who is feeling neglected in the society”- Fabian*

*“But then I had some kids who were on my neck quite disturbing me and making me feel like I'm a different being.”- Hana*

*“You know, a lot of people just use autism as an equivalent for like stupid or something… so many people look at autistic people as kind of just lost causes”- Grace*

However, creativity emerged as a pathway for participants to embrace their uniqueness and facilitate the affirmation of their own identities.

*“I am being an autistic person. It has affected me negatively. And being a creative person, it affects my life positively.”- Eddie*

*“I think being creative makes me feel special, happy. I'm proud of who I am. I feel like it is something I can hold on to. I feel like I have this gift. I think I mentioned that I feel creativity is like a gift to me.”- Brian*

In summary, creativity enabled our participants to view their identities positively, empowering them to overcome societal stigma and embrace their unique strengths.

## Discussion

The findings of our thematic analysis illustrate the complexity behind the relationship between neurodivergence and creativity, as perceived by neurodivergent people. They show how creative expression may not only serve as a medium for expression and emotional regulation but also as a source of strength and empowerment, enabling neurodivergent people to navigate and thrive within their unique experiences. Now we shall use our discussion to integrate our findings with existing research surrounding neurodivergence, to propose two hypotheses that might explain a relationship between neurodivergence and creativity. These hypotheses stem from the lived experience and perspectives of the 10 neurodivergent people who participated in this study, as described in the following sections.

### Hypothesis 1: Innate traits hypotheses

Within our ‘Innate Traits Hypothesis’ (Fig 2), we suggest that specific characteristics, believed to be innate to certain forms of neurodivergence, might facilitate the cultivation of creative abilities. In this study, participants frequently expressed a sense of thinking differently from the general population, a trait that resonates with the definition of neurodivergence— a mind that functions in a way which diverges significantly from the societal conceptions of ‘normal’ [5]. Divergent thinking, as coined by J.P Guilford [40], involves the capacity to generate creative ideas by synthesising diverse types of information in unconventional ways. Thus, if neurodivergent people demonstrate a propensity for thinking outside conventional frameworks, it stands to reason that they may excel in divergent thinking. A previous study exploring the association between divergent thinking and specific forms of neurodivergence found that ADHD was associated with heightened levels of divergent thinking, although such a link wasn't observed with autistic people [41]. However, the study in question employed sharp time constraints in its measure for divergent thinking [41], potentially disadvantaging autistic people, who often require additional time for cognitive processing [42]. Therefore, we argue that there is a necessity for additional research into the domain of divergent thinking and its association with neurodivergence, particularly within the autistic population – potentially employing alternative, more inclusive measures of divergent thinking, and led by neurodivergent researchers.

**Fig 2:**
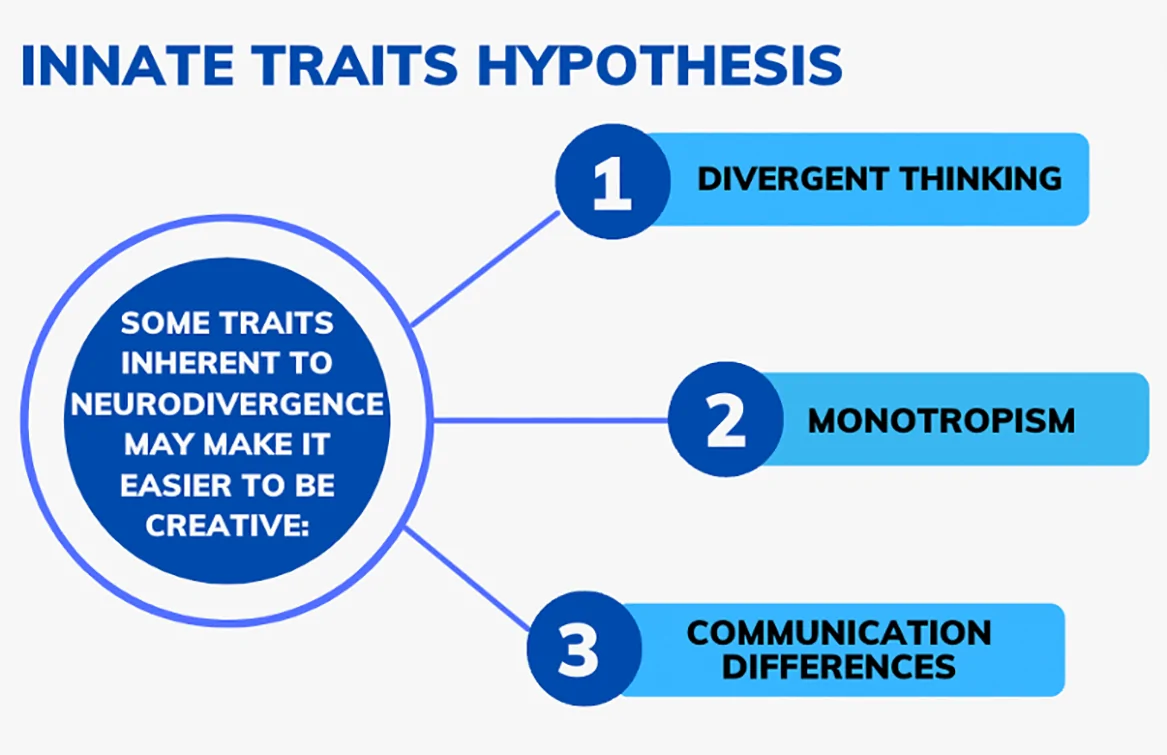
Innate traits hypothesis.

In addition, the theory of monotropism may also be relevant to the development of creative traits in neurodivergent people. Monotropism is an autistic-derived theory of autism, which asserts that autistic people have a narrow attentional focus on their special interests, which can be highly intense and relatively few in number [43,44]. This theory also involves the concept of “sticky attention,” indicating that autistic people may be slower to disengage from their special interests [44]. While monotropism is often discussed in relation to autism, some suggest it is also relevant to ADHD [45]. As observed in our own findings, some participants felt that their creativity allowed them to fully immerse themselves in their special interests, suggesting a key role for monotropism in their creative processes. A core aspect of monotropism is hyperfocus, which has long been poorly defined in scholarly literature, with concerted efforts to establish a precise definition only emerging in recent years [46]. Within the context of neurodivergence, hyperfocus has historically been depicted as a significant deficit, characterised by an inability to disengage from activities and a propensity to neglect other obligations [47,48]. However, contemporary research presents a re-evaluation of hyperfocus, suggesting that it may share conceptual similarities with the notion of ‘flow’, which is typically regarded in a positive light as an automatic, effortless, and intensely focused state [47]. Although no direct investigations have been conducted into the association between hyperfocus and creativity, one study has found a statistically significant relationship between flow and creativity [49]. Should the concepts of flow and hyperfocus indeed prove synonymous, this could potentially lay the groundwork for a hypothesis suggesting that the trait of hyperfocus, inherent in certain forms of neurodivergence, is linked to creative attributes.

Furthermore, a significant number of our participants emphasised their inclination for expressing themselves through creative mediums rather than through social communication. Understanding the underlying reasons for this phenomenon presents a challenge due to a scarcity of research in this area. One possible explanation is that neurodivergent people may cultivate a talent for creative expression in response to the isolation and stigma imposed by their distinct social communication methods. Another factor may arise from a potential neurodevelopmental angle, wherein neurodivergent people possess distinct methods of communication that may facilitate expression through creative mediums. Further investigation is warranted to understand this relationship between social communication variances and creativity in this context. Exploring this line of research has the potential to further challenge deficit-based interpretations of social communication, particularly within the autistic population, which are already being questioned [50].

### Hypothesis 2: Social isolation hypothesis

Our second hypothesis for understanding the relationship between neurodivergence and creativity places social isolation at its core (Fig 3). Each of our 10 participants in this study shared personal narratives of enduring social isolation from childhood into adulthood, despite many expressing a desire for social connection. These individual experiences resonate with broader research indicating that neurodivergent people face a higher risk of social isolation compared to the general population [18,51]. This isolation is likely due to the significant discrimination and prejudice that neurodivergent people face [36]. The negative impacts of social isolation on mental and physical health are clear, as demonstrated by the marked declines observed in the general population during the COVID-19 pandemic [52]. The decline in health resulting from social isolation extends to neurodivergent populations. Contrary to prevailing misconceptions, many neurodivergent people strongly desire social interaction [53]. Furthermore, autistic and ADHD people may be more susceptible to the adverse physical and psychological consequences of loneliness [19,54]. In essence, neurodivergent people may not only be at elevated risk of social isolation but may also be more prone to the detrimental effects of loneliness.

**Fig 3:**
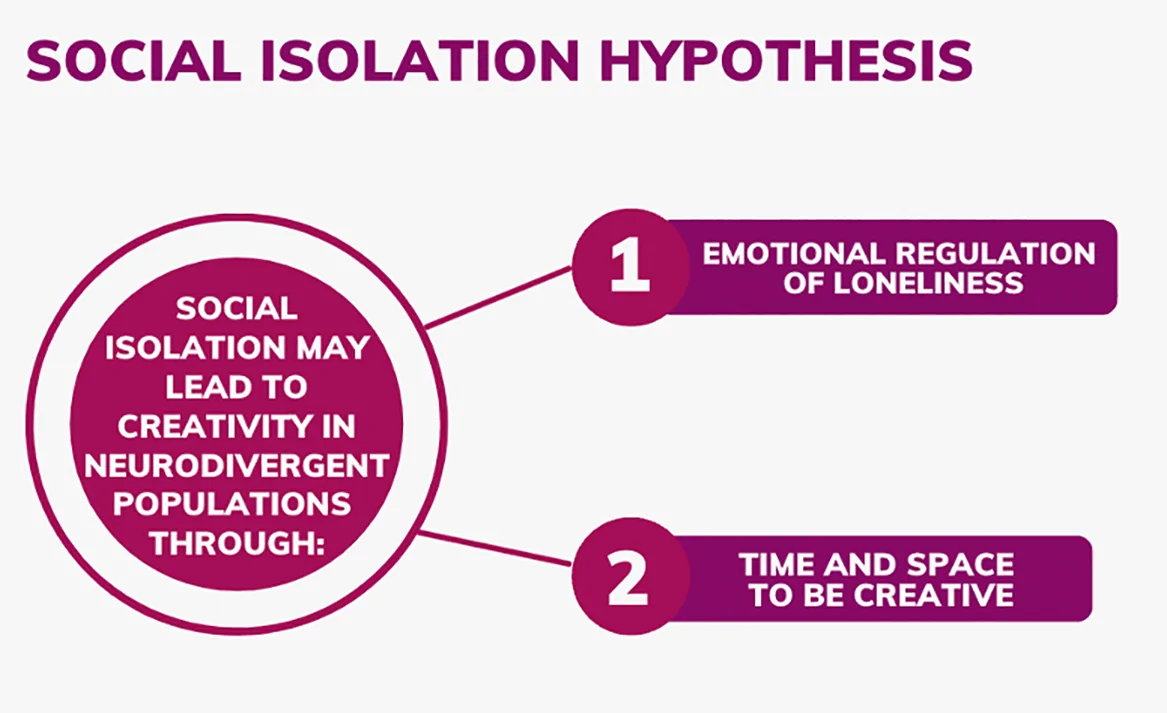
Social isolation hypothesis.

Some of our participants in this study suggested a potential connection between their experiences of social isolation and the development of their creative abilities. Although research on the effects of isolation on creativity within neurodivergent populations is lacking, the COVID-19 pandemic offered a relevant context for exploring this relationship in the general population. This pandemic resulted in unprecedented levels of loneliness across societies, and studies indicate that engaging in creative activities might alleviate these feelings [55]. This potential link could be attributed to the role of creativity in emotional regulation – as some participants implied, engaging in creative activities could offer solace from the emotional distress associated with loneliness. This aligns with existing research on the relationship between creativity and loneliness, suggesting that creativity serves as a constructive mechanism for managing feelings of loneliness [53]. Creativity may engender feelings of mastery and empowerment which has benefits for overall well-being [56]. Furthermore, creativity seemed to empower certain participants to view their social isolation in a more positive context. Their sense of solitude afforded them the time and space to reflect on their experiences and channel them through creative mediums. Creativity seemed to also facilitate a deeper appreciation of their own worth. In a societal landscape that marginalises them [16], creativity serves as a means for these people to assert their identity proudly and find a sense of belonging. Hence, we advocate for additional exploration of the connection between creativity and social isolation among neurodivergent communities.

### Strengths & limitations

This study presents several strengths and limitations that warrant reflection. While qualitative methods are particularly well-suited for exploring the nuanced relationship between neurodivergence and creativity, they do not allow for generalisability. Additionally, the methodology limits the dependability of the findings, as they are not representative of all neurodivergent people. However, the study’s philosophical stance does not aim for generalisability or dependability. Instead, it acknowledges the inherently subjective and nuanced nature of both neurodivergence and creativity. From this perspective, the study’s strength lies in its recognition of the subjectivity embedded within these concepts, offering a deeper understanding grounded in lived experience. The use of qualitative methods enables insight into the personal experiences of neurodivergent adults, hearing voices that are often marginalised and providing space for new ways of understanding creativity. As the first qualitative investigation of this specific relationship, this study lays foundations that future research might build upon using quantitative methods.

It is also essential to reflect on credibility and confirmability. During thematic analysis, BE actively reflected on his positionality and potential biases, aiming to prioritise participants’ perspectives, an approach compatible with critical realism. However, some degree of unconscious bias is inevitable, which impacts confirmability. This was mitigated by the involvement of a second coder (LQ), thereby enhancing the credibility of the findings.

In addition, the absence of a comparison group is consistent with the aims and qualitative exploratory design of this study. Rather than comparing creativity between neurodivergent and neurotypical people, the study aimed to centre the perspectives of neurodivergent people and explore how they understand and experience creativity in relation to their neurodivergence. By focusing on lived experiences and self-perceptions, this approach provides qualitative insight into a topic that may not be adequately captured through standardised measures of creativity. As such, this design represents a methodological strength, enabling a deeper understanding of participants’ perspectives.

Another important consideration lies in the participant selection criteria. Taking into account the subjective nature of neurodivergence, the inclusion criteria were kept broad, allowing anyone with a formal diagnosis of neurodivergence to participate. While focusing on a single form of neurodivergence, such as autism, could yield more specific findings, given the evolving nature of diagnostic labels, where definitions shift over time, and individuals may be re-diagnosed or de-diagnosed, we argue that a broader approach is warranted. Many neurodivergent individuals experience overlapping traits and co-occurring conditions [57], making strict categorisation challenging. By including participants with diverse neurodivergent experiences, we aim to capture a more holistic understanding of creativity in this population. However, our choice of requiring a formal diagnosis to participate is also worth reflection. This aligns well with the critical realist stance of this study, facilitating consistency and reducing potential bias. However, as discussed earlier, many neurodivergent individuals face substantial barriers to obtaining a formal diagnosis, including lengthy waiting times, limited access to appropriate assessment services, financial barriers associated with private assessments, and fear or mistrust of healthcare services stemming from previous negative experiences [14,15]. This study did not include these voices, which is an important limitation to consider.

Furthermore, our criteria used to measure creativity were far from objective, which is an important limitation to consider in terms of transferability. Enrolment in a creative degree does not equate to being inherently creative, nor does it represent the diverse ways creativity manifests. Many creative individuals do not pursue creativity academically. However, we made this decision to recruit those with a creative academic background since these people would likely have more to say about this relationship. However, this also introduces a degree of selection bias with these individuals likely to perceive a close relationship between neurodivergence and creativity. In addition, we argue that seeking an objective measure of creativity in this context would be both challenging and potentially reductive. Current measures of creativity have limited validation with neurodivergent populations and may not fully capture the unique expressions of creativity among them. Our study challenges the notion that creativity can be measured universally or objectively, asserting instead that creativity is deeply personal and context dependent. From a critical realist perspective, while an objective reality exists, social and personal influences shape how creativity is perceived and performed. Given the challenges in measuring the relationship between neurodivergence and creativity using objective measurements [30], we argue that the approach to measuring creativity in this study represents a necessary and appropriate shift.

Lastly, while the lack of detailed demographic data may be seen as a limitation, this was a deliberate ethical choice. Given the sensitive and personal nature of the narratives shared, preventing participant triangulation and maintaining anonymity was a priority. In line with principles of relational ethics, we argue that safeguarding trust and confidentiality in the researcher-participant relationship outweighed the potential benefit of more detailed demographic data.

## Conclusions

The results of this study shed light on two hypotheses regarding the correlation between neurodivergence and creativity. The innate trait hypothesis proposes that specific inherent traits within neurodivergent people may predispose them to heightened creativity, while the social isolation hypothesis suggests that the increased isolation experienced by neurodivergent populations could also foster creativity. There is a likelihood that these hypotheses are both valid and potentially intersecting. It is highly likely that there are a plethora of additional reasons behind the potential relationship behind neurodivergence and creativity, with creativity being a nuanced and complicated concept. However, these hypotheses deeply underscore the importance of considering the neurodiversity paradigm. Our results highlight that some neurodivergent traits, when placed within a creative context, can become strengths. This highlights the possibility that the pathologisation of neurodivergent traits is not just damaging but also invalid, with the reality being much more nuanced. Furthermore, challenging notions of the ‘normal’ brain should continue within this field of research and there should be further acknowledgement of the rich spectrum of diversity within the human mind, all of which with their unique strengths and challenges. By doing so, a more inclusive and accepting society can be formed that embraces all the different colours of the mind.

## Supporting information

S1 Appendix Recruitment poster. (PNG)

S2 Appendix Interview topic guide. (DOCX)
