# Supplementary material for: Colours of the mind: A qualitative study exploring the relationship between neurodivergence and creativity
Source: PLoS One. 2026 Oct 28;21(10):e0359079. doi: 10.1371/journal.pone.0359079 (PMC13716226; doi:10.1371/journal.pone.0359079)
Supplement: Supplementary file 1 [file pone.0359079.s002.docx]

***Interview topic guide***

*Note: The interviews were semi-structured and so the interviews would often explore other avenues not outlined here at the participants request.*

- Introductions
  - Introduce myself and what drew me to creating this project.
  - Offer any adjustments for the interview to make the experience more comfortable for them.
  - Ask the participant to introduce themselves and ask some ice breaker questions eg what are they studying, what year are they in, how are they finding their course.
- Creative background
  - What is their background in creativity? How long have they been creative for? Where was their passion from?
- Neurodivergence
  - How do they relate to their neurodivergence? Do they view their neurodivergence as positive or negative and how has this changed during their life?
- Neurodivergence and creativity
  - Do they think that there is a relationship between being neurodivergent and being creative? Why?
  - Do they think, based on their experience, that neurodivergent people are more creative than the general population?
  - Do they think their neurodivergence contributes to their creativity? If so, why?
  - What is their creative process and how does their neurodivergence affect this?
  - How does their creativity affect the way they view their neurodivergent identity?
- Accessibility and adjustments of creative pathways
  - What drew them to a creative degree?
  - How accessible is a creative degree to them?
- Conclusion
  - Is there anything else you would like to add?
